# A coarse-grained red blood cell membrane model to study stomatocyte-discocyte-echinocyte morphologies

**DOI:** 10.1371/journal.pone.0215447

**Published:** 2019-04-19

**Authors:** Nadeeshani Maheshika Geekiyanage, Marie Anne Balanant, Emilie Sauret, Suvash Saha, Robert Flower, Chwee Teck Lim, YuanTong Gu

**Affiliations:** 1 School of Chemistry, Physics and Mechanical Engineering, Queensland University of Technology (QUT), Brisbane, Queensland, Australia; 2 Research & Development, Australian Red Cross Blood Service, Brisbane, Queensland, Australia; 3 University of Technology Sydney (UTS), Ultimo, New South Wales, Australia; 4 Department of Biomedical Engineering, Faculty of Engineering, National University of Singapore, Singapore; 5 Biomedical Institute for Global Health Research and Technology, National University of Singapore, Singapore; 6 Mechanobiology Institute, National University of Singapore, Singapore; University of Michigan, UNITED STATES

## Abstract

An improved red blood cell (RBC) membrane model is developed based on the bilayer coupling model (BCM) to accurately predict the complete sequence of stomatocyte-discocyte-echinocyte (SDE) transformation of a RBC. The coarse-grained (CG)–RBC membrane model is proposed to predict the minimum energy configuration of the RBC from the competition between lipid-bilayer bending resistance and cytoskeletal shear resistance under given reference constraints. In addition to the conventional membrane surface area, cell volume and bilayer-leaflet-area-difference constraints, a new constraint: total-membrane-curvature is proposed in the model to better predict RBC shapes in agreement with experimental observations. A quantitative evaluation of several cellular measurements including length, thickness and shape factor, is performed for the first time, between CG-RBC model predicted and three-dimensional (3D) confocal microscopy imaging generated RBC shapes at equivalent reference constraints. The validated CG-RBC membrane model is then employed to investigate the effect of reduced cell volume and elastic length scale on SDE transformation, to evaluate the RBC deformability during SDE transformation, and to identify the most probable RBC cytoskeletal reference state. The CG-RBC membrane model can predict the SDE shape behaviour under diverse shape-transforming scenarios, in-vitro RBC storage, microvascular circulation and flow through microfluidic devices.

## Introduction

Red blood cell (RBC) is a unique cell without any nucleus or mitochondria [1–3] and is remarkably simple in its structure. The most important function of RBC is the transfer of oxygen to body tissues and RBC is adapted with many features to maximize its performance as a gas carrier. It is extremely deformable and elastic to sustain its passage through narrow capillaries of the microvasculature [3, 4]. RBC holds ~ 40% excess surface area as compared to a sphere with the same volume [2, 5], and the higher surface area allows an increased gas transfer across its surface [6]. The structural integrity and the stability of the RBC is due to its membrane which is comprised of three main components; the phospholipid bilayer, transmembrane proteins and the cytoskeleton [4, 7]. The cytoskeleton is mainly composed of spectrin tetramers connected at actin junctional complexes in a triangular network form [7, 8], and is the major contributor to the highly elastic nature of the RBC, whereas the lipid bilayer contributes to the viscosity and area preserving nature of the RBC [9, 10].

**Fig 1:**
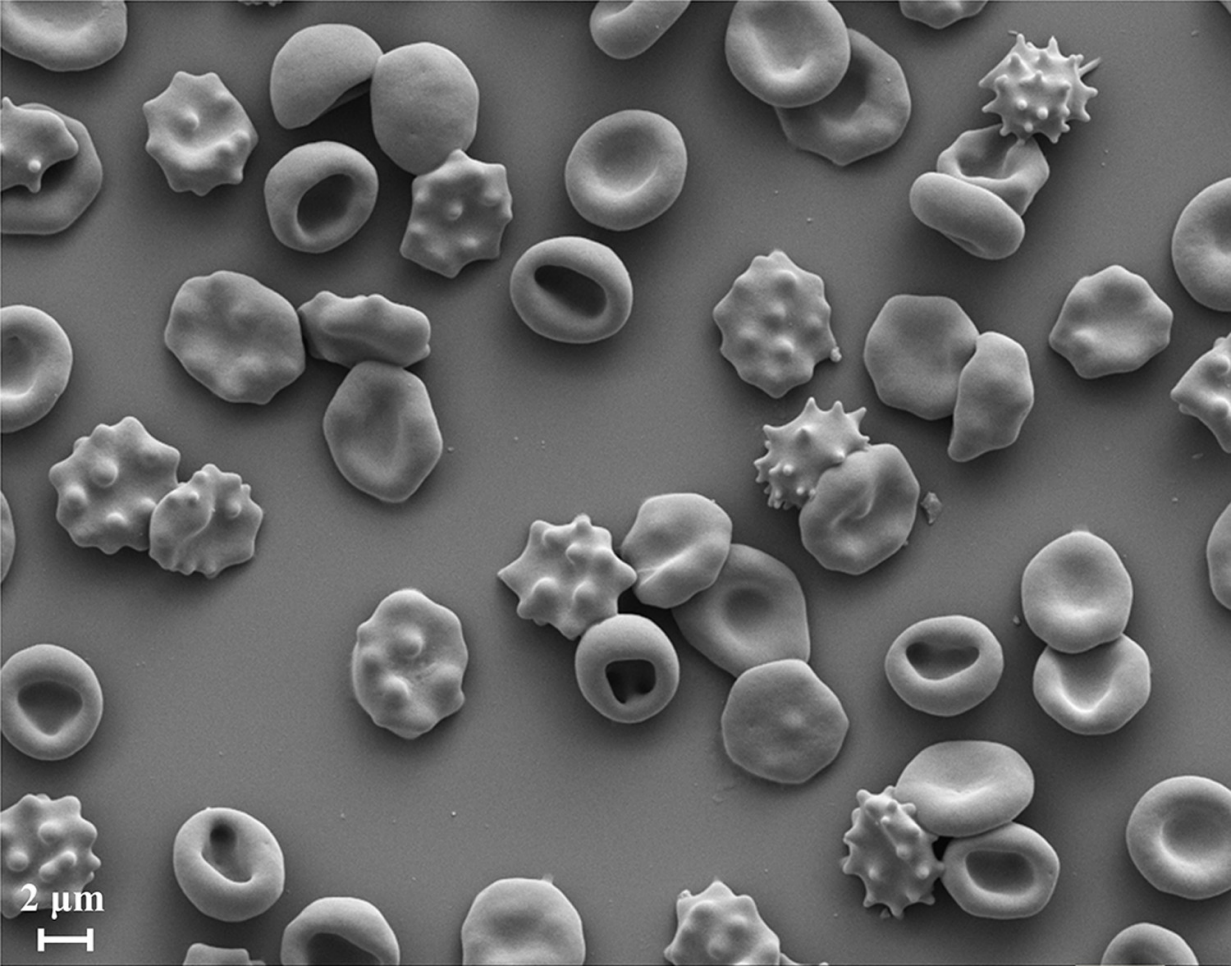
Scanning electron microscope (SEM) image of a RBC population having different morphology stages of stomatocytosis and echinocytosis.

It has been found that the normal biconcave shape of RBC can be modified into stomatocytes and echinocytes through variety of agents, some of them such as amphipaths, extracellular ionic strength and pH, causing reversible transformations at constant membrane surface area and cell volume [11–16]. Stomatocytes are cup-like concave shapes whereas echinocytes are crenated shapes with a spiculated cell surface [9, 12–15, 17]. Stomatocytes and echinocytes shapes are further categorized into subclasses I, II, III and IV considering different stages of concavity and crenation. The Bessis’ nomenclature is commonly used to identify different RBC shapes [18], and the different stages of stomatocyte-echinocyte can be described as follows. Stomatocyte I is a cup shape with a shallow circular invagination; Stomatocyte II is a cup shape with a deeper invagination, still at least approximately circular; Stomatocyte III is a cup shape with a deep invagination, often elongated into a mouth-like slit and sometimes accompanied by other pit-like invaginations; Sphero-stomatocyte (Stomatocyte IV) is a spherical shape with small interior buds still attached to the membrane; Echinocyte I is a disc with several undulations around its rim; Echinocyte II is a flattened elliptical body with rounded spicules distributed more or less uniformly over its surface; Echinocyte III is an ovoid or spherical body with sharper and more numerous (30–50) spicules distributed evenly over its surface; and Sphero-echinocyte (Echinocyte IV) is a sphere with small sharp projections still attached to its surface [19]. Refer to Fig 1 for a representation of RBC morphology at different stages of stomatocytosis and echinocytosis.

Among the identified many physical and chemical conditions, cationic amphipaths, low salt, low pH and cholesterol depletion can transform normal biconcave RBC into stomatocytes whereas anionic amphipaths, high salt, high pH, adenosine triphosphate (ATP) depletion, cholesterol enrichment, and proximity to a glass surface can transform normal biconcave RBC into echinocytes [12, 20]. These shape changes are reversible to the stage where sphero-stomatocyte and sphero-echinocyte become smooth spheres when small interior buds and vesicles are pinched off from the RBC surface [17]. These reversible RBC shape changes from biconcave shape to-and-from stomatocyte and echinocyte shapes are widely known as stomatocyte-discocyte-echinocyte (SDE) shape transformations. SDE shape transformations have a universal nature for the resultant shape and do not depend on the engaged shape-transforming agent [12].

Sheetz and Singer [21] proposed the widely known bilayer couple hypothesis to explain SDE transformation, and according to the bilayer-couple hypothesis, the two leaflets of the membrane bilayer responds differently to various perturbations and remain coupled to each other. Therefore, the expansion of the outer bilayer-leaflet relative to the inner bilayer-leaflet due to any shape-transforming agent results in convex forms on the membrane surface and induces echinocytes. Similarly, the expansion of the inner bilayer-leaflet relative to the outer bilayer-leaflet due to any shape-transforming agent results in concave forms on the membrane surface and induces stomatocytes. In this manner, the universal nature of SDE transformation is explained through the bilayer-couple hypothesis, and any change in relaxed area-difference between bilayer-leaflets is the necessary and sufficient factor for shape changes.

Several research studies have studied the SDE transformation [12, 13, 15, 17, 20, 22–34], and here, we discussed the development of an improved computational approach to accurately predict SDE transformation. Following subsections briefly introduce the computational approaches on RBC simulations in general, the bending energy models for SDE prediction, and discuss the few computational simulations on SDE transformation.

### Computational simulations of RBC

Computational modelling and experimental studies have been carried out to investigate the biomechanical and rheological behaviour of RBC [9, 35–40]. Due to the simplicity of the RBC structure, they can be numerically approximated as a bag of concentrated haemoglobin solution surrounded by a thin macroscopically homogeneous membrane [13]. The thickness of the lipid bilayer being ~ 4 nm [28] and the offset between lipid bilayer-cytoskeleton being ~ 30–40 nm [28], RBC membrane can be treated as a two-dimensional (2D) viscoelastic surface in a three-dimensional (3D) space in the cellular length scale [9, 13]. The heterogeneous nature of the RBC membrane can be reasonably approximated as homogeneous in its properties for length scales above 100 nm [13, 41], and is suitable for mesoscopic and macroscopic scale investigations. However, a more realistic and accurate RBC representation requires detailed description of its structure, and therefore the incorporation of the properties of lipid bilayer, cytoskeleton and transmembrane proteins. There are several types of computational modelling and simulation approaches for RBC studies, and the prime approaches are continuum, particle-based and hybrid continuum-particle [41] based techniques. RBC membrane and associated fluid components are considered as homogeneous material under continuum approach, whereas the particle-based modelling represents these components via a network of springs and particulate assembly [42], respectively. It is difficult for the continuum approaches to incorporate the detailed structure of RBC membrane, whereas particle-based methods are capable of detailed representation of the RBC membrane but require high computational cost to represent it at the molecular level through molecular dynamics approaches. Therefore, particle-based modelling approach in macroscale and mesoscale are widely used to study RBC behaviour. Besides, hybrid continuum-particle technique is the merging between continuum and particle-based approaches and can lead to more accurate and computationally efficient numerical simulations to study a broad range of biochemical and rheological behaviour of RBC.

For computational simulation purposes, the equilibrium RBC shape was derived by minimizing the in-plane shear energy and the out-of-plane bending energy under the reference constraints of cell membrane area and cell volume [9, 42]. There are several forms of Helfrich energy; spontaneous curvature model (SCM), bilayer coupling model (BCM), and area-difference-elasticity (ADE), which have been used to describe the RBC membrane bending energy [9, 23]. The SCM describes the membrane bending energy ($E_{Bending}^{SCM}$) for a membrane with surface area, A and local bending modulus,κ, such that [23, 24];

$$E_{Bending}^{SCM}\;\left[{\text{C}}_{\text{1}}{,\text{C}}_{\text{2}}\right]\text{=}\;\frac{\kappa }{\text{2}}\;\oint \text{dA}\;{\left({\text{C}}_{\text{1}}\left(\text{r}\right){\text{+ C}}_{\text{2}}\left(\text{r}\right)\text{-}\;\bar{{\text{C}}_{\text{0}}}\right)}^{\text{2}}$$ (1)

where, $C_{1}(r)$ and $C_{2}(r)$ are the principal curvatures at the point r on the membrane surface, and $\bar{C_{0}}$ is the spontaneous curvature. $\bar{C_{0}}$ indicates any asymmetry between the two bilayer-leaflets. The SCM model derived, vesicle shape is obtained at the minimum of $E_{Bending}^{SCM}$ for given A and vesicle volume, V.

The BCM is based on the bilayer-couple hypothesis, and assumes fixed area for a membrane lipid molecule and no molecular exchange between the two bilayer-leaflets. Therefore, the area of each bilayer-leaflet remains constant, and the area-difference between the two bilayer-leaflets (ΔA) can be determined from the integrated mean curvature over the membrane surface, such that [23];

$$\Delta A=D\;\oint dA\;\left(C_{1}\left(r\right)+\;C_{2}\left(r\right)\right)$$ (2)

where, D is the distance between bilayer leaflets. The membrane bending energy $E_{Bending}^{BCM}$ in this instance is determined for a defined reference surface with fixed ΔA as another constraint, such that [23];

$$E_{Bending}^{BCM}\;\left[C_{1},\;C_{2}\right]=\;\frac{\kappa }{2}\;\oint dA\;{\left(C_{1}\left(r\right)+\;C_{2}\left(r\right)\right)}^{2}$$ (3)

The BCM model derived vesicle shape is obtained at minimum $E_{Bending}^{BCM}$ for given A,V and ΔA. It has been proven elsewhere [24, 27, 43] that both SCM and BCM models lead to same shape equations, and the vesicle shape behaviour is an extensively studied aspect [13, 15, 19, 23, 24, 27, 28, 31, 33, 34, 43–49]. The ADE model is a combined representation of SCM and BCM, and the ADE model determined membrane energy ($E_{ADE}$) for a vesicle having A,V and ΔA is as follows [23],

$$E_{ADE}\;\left[C_{1},\;C_{2},\;\Delta A\right]=\;\frac{\kappa }{2}\;\oint dA\;{\left(C_{1}\left(r\right)+\;C_{2}\left(r\right)-\;\bar{C_{0}}\right)}^{2}+\;\frac{\bar{\kappa }}{2}\;\frac{\pi }{A\;D^{2}}\;{\left(\Delta A-\;\Delta A_{0}\right)}^{2}$$ (4)

where, $\bar{\kappa }$ is the non-local bending modulus and $\Delta A_{0}$ is the reference area-difference between bilayer-leaflets. The ADE model converges to SCM model at $\;\bar{\kappa }/\kappa \;\to \;0$, and into BCM at $\;\bar{\kappa }/\kappa \;\to \;\infty$ [23, 46, 48]. The discocyte RBC shape is usually derived from SCM approach at $\bar{C_{0}}\;\sim \;0\;$[50], and the following approaches have been followed to predict the SDE transformation.

### Computational simulations of SDE transformation

The shape, behaviour and deformability of a RBC have been extensively studied through computational simulation methods [9, 35, 36] for various conditions and scenarios. However, despite the numerous experimental and theoretical investigations on RBC shape transformations, the numerical predictions on full SDE transformations of RBC are limited [15, 47]. The existing limited number of numerical shape predictions use ADE membrane bending model to represent the bending resistance of the lipid-bilayer, and no information of the use of SCM and BCM in numerical implementations of SDE shape predictions are available. Nevertheless, the coarse-grained (CG)-RBC membrane model is developed on BCM approach and demonstrates the potential of BCM approach to accurately predict SDE transformation.

The studies by Lim et al. [19, 28, 49], and Khairy and Howard [31, 33] have used a continuum numerical approach based on ADE model to predict different stages of stomatocytes and echinocytes of SDE transformations. These studies have used a single parameter; effective-spontaneous-curvature as the control parameter to predict the different stages of SDE transformation under the constraints of membrane surface area and cell volume. The effective-spontaneous-curvature is a combination of both spontaneous membrane curvature and the reference area-difference of bilayer-leaflets, and at different values of effective-spontaneous-curvature, the corresponding RBC shape has been determined. It has also been identified that the effects of both spontaneous membrane curvature and bilayer-leaflet-area-difference are present in RBC membrane, and their effect is comparable in magnitude [19, 31]. Furthermore, any lipid asymmetry can be cancelled out by manipulating the bilayer-leaflet-area-difference, while any bilayer-leaflet-area-difference can be cancelled out by manipulating the lipid asymmetry of the lipid bilayer. Therefore, the spontaneous membrane curvature and the area-difference of bilayer-leaflets can be combined, and since, the spontaneous membrane curvature and the bilayer-leaflet-area-difference cannot yet be measured directly, it is also convenient to employ their combined effect through a single control parameter. However, the use of κ and $\bar{\kappa }$ in the same scale could lead to a significant difference [11] between the reference spontaneous membrane curvature and bilayer-leaflet-area-difference with the instantaneous membrane curvature and bilayer-leaflet-area-difference at the equilibrium. Furthermore, a detailed RBC membrane representation is required to study RBC shape transformations in the presence of membrane inhomogeneity, defective cytoskeleton and blood disorders that affect the membrane structure [8] such as hereditary-spherocytosis (HS) and hereditary-elliptocytosis (HE) [51, 52]. Therefore, the studies by Lim et al. [19, 28, 49], and Khairy and Howard [31, 33] are difficult to be extended to study the RBC morphology at above diseased conditions.

Chen and Boyle [15] have also developed a CG based spring-particle (SP) numerical approach to predict the SDE transformations, and have suggested a surface curvature approximation that is independent of the network topology to estimate the membrane bending energy. Their ADE model is comprised of the two distinct parameters; spontaneous membrane curvature and reference area-difference between bilayer-leaflets. However, the spontaneous membrane curvature and the reference area-difference between bilayer-leaflets, both being mathematical relationships of the membrane curvature, these control parameters for SDE transformation can be combined together as in the studies by Lim et al. [19, 28, 49], and Khairy and Howard [31, 33]. In addition, Chen and Boyle [15] have proposed shape boundaries for a wide range of RBC cell volume and membrane-area-difference. For all these SDE shape predictions; Lim et al. [19, 28, 49], Khairy and Howard [31, 33] and Chen and Boyle [15], the minimum energy configuration of the RBC membrane has been determined by the in-plane elastic energy and out-of-plane bending energy, under the constraints on cell surface area and cell volume. Lim et al. [19, 28, 49], and Khiary and Howard [31, 33] have both employed the non-linear Skalak law to estimate the in-plane shear deformation of the RBC membrane. Chen and Boyle [15], in their CG approach, have adopted the worm-like-chain (WLC) type springs to estimate the resulting elastic energy between these CG particles. The membrane bending energy is estimated through discrete approximations of Helfrich’s energy for membrane triangulation.

These computational simulation approaches qualitatively agree well with the experimental observations of RBC SDE transformation. However, all these studies have used the ADE model in which $\;\bar{\kappa }\;/\kappa \;\approx 1.0$, and the actual bilayer-leaflet-area-difference at the equilibrium differs from the reference conditions. Therefore, it is difficult to quantitatively compare numerical predictions against the experimental observations as the experimentally extracted bilayer-leaflet-area-difference is at the equilibrium. In addition, it is difficult to find any implementation of SCM method to successfully predict discocyte-echinocyte transformation. Therefore, it is reasonable to expect the presence of any bilayer-leaflet-area-difference with $\;\bar{\kappa }\;/\kappa \;\neq 1.0\;$ in order to predict discocyte-echinocyte transformation. Furthermore, if $\bar{\kappa }\;/\kappa \;\gg 1.0$ as in the case of BCM, then the deviation of the equilibrium bilayer-leaflet-area-difference form the reference conditions would be minimal. By this means, a framework to quantitatively validate the developed computational simulations against experimentally observed SDE transformation can be achieved, and will enhance the applicability of numerical predictions to investigate exact SDE transformation conditions. The universal nature of SDE transformation in the presence of echinocytic and stomatocytic shape-transforming agents can be adapted to suit distinctive scenarios if independent measurements of control parameters are available experimentally or computationally. Therefore, a CG-RBC membrane model inspired from the previous research by Lim et al. [19, 28, 49], is proposed with enhanced applicability to address some of the above concerns, namely, the significant difference between effective-spontaneous-curvature and instantaneous membrane curvature and bilayer-leaflet-area-difference at the equilibrium for ADE model, and the lack of framework to quantitatively validate the developed computational simulations against experimentally observed SDE transformation. The proposed improved CG-RBC membrane model predicts the SDE transformation and is first validated against experimentally observed RBC shapes. It is then applied to study the effect of reduced cell volume and elastic length scale on SDE transformation. Furthermore, the deformability of different stages of SDE transformation and the most probable cytoskeletal reference state of the RBC are investigated using the present CG-RBC membrane model.

## Methods

The RBC membrane is the primary contributor for its mechanical nature since a RBC does not possess any internal structure. The lipid-bilayer contributes to its out-of-plane bending resistance and surface area incompressibility while the cytoskeletal spectrin network contributes to the in-plane shear deformation [53], and its cytoplasm contributes to the volume incompressibility of the RBC. The Helmholtz free energy of the RBC membrane is the collective contribution of out-of-plane membrane bending energy, in-plane shear energy and the energy penalty due to cell surface area and volume constraints relative to the specified reference membrane configuration. The equilibrium RBC shape is determined at the minimum free energy state of the RBC membrane.

### Free energy of the CG-RBC membrane model

The developed CG-RBC membrane model employs $N_{V}$ particles to represent the actin junctional complexes of the RBC membrane and form a 2D triangulated surface of $N_{t}$ triangles. The $N_{S}$ adjacent particle-particle connections of the triangulated surface represent the cytoskeletal spectrin links. The in-plane shear energy of the RBC membrane $\left(E_{Stretching}\right)$ is estimated based on the coarse-graining approach implemented by Fedesov et al. [50], and is composed of an attractive potential $\left(E_{WLC}\right)$ in the form of the WLC potential and a repulsive potential $\left(E_{POW}\right)$ in the form of a power function. $E_{Stretching}\;$ can be expressed as follow [50];

$$E_{Stretching}\;\left(l_{j}\right)=\;\sum_{j\;\epsilon \;1\ldots N_{S}}\left[E_{WLC}\left(l_{j}\right)+\;E_{POW}\left(l_{j}\right)\right]$$ (5)

$$E_{WLC}\left(l_{j}\right)=\;\frac{k_{B}\;T\;l_{max}}{4\;p}\;\frac{3x_{j}^{2}-\;2x_{j}^{3}}{1-\;x_{j}}$$ (6)

$$E_{POW}\left(l_{j}\right)=\;\{\begin{array}{cc} \frac{k_{p}}{\left(m-1\right)\;l_{j}^{m-1}}, & m\;\neq 1\\ -\;k_{p}\;log\left(l_{j}\right), & m=1 \end{array}$$ (7)

where, $l_{j}$ is the length of $j^{th}$ link, $k_{B}$ is the Boltzmann constant, T is the absolute temperature, $l_{max\;}$ is the maximum link extension, p is the persistence length, $k_{p}$ is the power function coefficient, and m is an exponent such that $m\;>0.\;x_{j}$ is defined as $x_{j}\;=\;l_{j}\;/{\;l}_{max}$. The experimentally estimated RBC membrane shear modulus $\left(\mu_{0}\right)$ lies between 4–12 μNm^-1^ [9, 50], and can be expressed as following for the CG-RBC membrane model [50]:

$$\mu_{0}=\;\frac{\sqrt{3}\;k_{B}\;T}{4\;p\;l_{max}\;x_{0}}\;\left(\frac{x_{0}}{2\;{\left(1-\;x_{0}\right)}^{3}}-\;\frac{1}{4\;{\left(1-\;x_{0}\right)}^{2}}+\;\frac{1}{4}\right)+\;\frac{\sqrt{3}\;k_{p}\;\left(m+1\right)}{4\;l_{0}^{m\;+\;1}}$$ (8)

where, $l_{0}$ is the equilibrium length of spectrin link and defined as $x_{0}\;=\;l_{0}\;/\;l_{max}$. The parameters ${\;k}_{p}$ and p can be estimated for a given $\mu_{0}$ and $x_{0}$ using Eqs (5) and (8) at the equilibrium state of specified cytoskeletal reference state.

The out-of-plane bending energy of the RBC membrane $\left(E_{Bending}\right)$ is estimated based on a discrete approximation of the Helfrich energy model [54] for a zero spontaneous membrane curvature, such that:

$$E_{Bending}=2\;\kappa \;\sum_{j\;\epsilon \;1\ldots \;N_{s}}\frac{M_{j}^{2}}{\Delta A_{j}}$$ (9)

**Fig 2:**
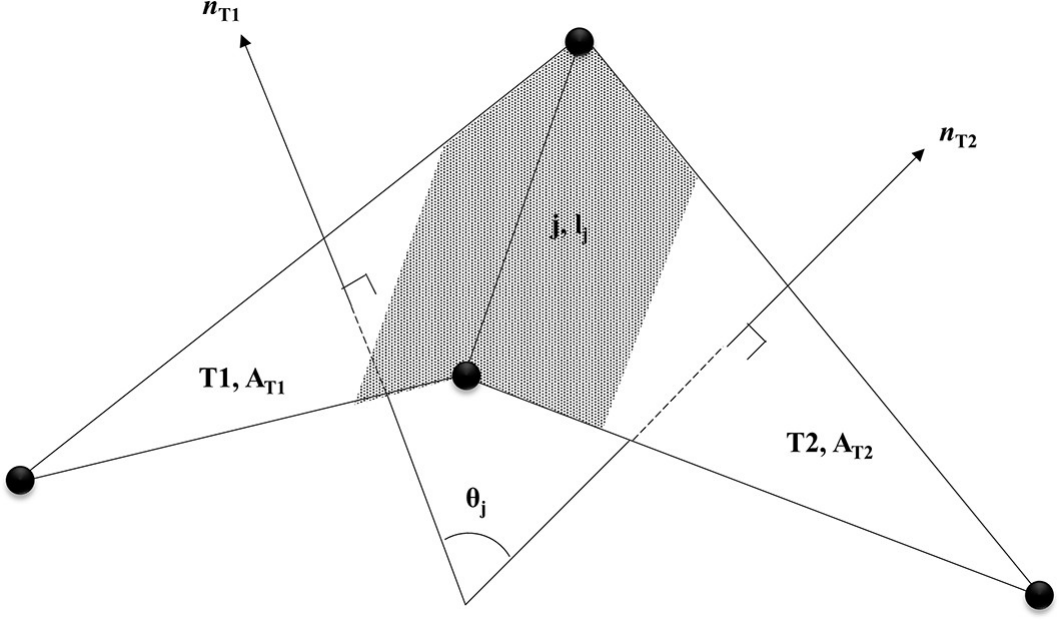
Illustration of $M_{j}$ at $j^{th}$ link. The shaded planer area is the area associated with the $j^{th}$ link. $n_{T1}\;$ and $n_{T2}$ are the normal vectors to the triangles T1 and T2 respectively.

$M_{j}$ is the membrane curvature at the triangle-pair that shares the $j^{th}$ link, and $\Delta A_{j}$ represents the surface area associated with the $j^{th}$ link. $M_{j}$ and $\Delta A_{j}$ can be given as:

$$M_{j}=\;\frac{1}{2}\;l_{j}\;\theta_{j}$$ (10)

$$\Delta A_{j}=\;\frac{1}{3}\;\left(A_{T1}+\;A_{T2}\right)$$ (11)

where $\theta_{j}$ is the angle between outward normal vectors to the triangles sharing $j^{th}$ link, and $A_{T1}$ and $A_{T2}$ are the planer area of T1 and T2 triangles respectively that share the $j^{th}$ link. The concave arrangement of a triangle-pair results in positive $M_{j}$ whereas the convex arrangement of a triangle-pair results in negative $M_{j}$. An illustration of the triangle-pair made of T1 and T2 triangles that shares the $j^{th}$ link is provided in Fig 2.

The energy components due to RBC surface area constraint $\left(E_{Surface\;Area}\right)$ and the cell volume constraint $\left(E_{Volume}\right)$ are estimated as follows [50, 53]:

$$E_{Surface\;Area}=\;\frac{1}{2}\;k_{A}\;{\left(\frac{A-\;A_{0}}{A_{0}}\right)}^{2}\;A_{0}+\;\sum_{k\;\in 1\ldots N_{t}}\frac{1}{2}\;k_{a}\;{\left(\frac{A_{k}-\;A_{k,0}}{A_{k,0}}\right)}^{2}\;A_{k,0}$$ (12)

$$E_{Volume}=\;\frac{1}{2}\;k_{v}\;{\left(\frac{V-\;V_{0}}{V_{0}}\right)}^{2}\;V_{0}$$ (13)

where, $A_{0}$ is the reference membrane surface area, A is the instantaneous membrane surface area, $A_{k,0}$ is the reference area of $k^{th}$ triangle, $A_{k}$ is the instantaneous area of $k^{th}$ triangle, $V_{0}$ is the reference cell volume and V is the instantaneous cell volume. $k_{A},\;k_{a}$ and $k_{v}$ represent the total surface area, local surface area and volume constraint coefficients, respectively. The resistance of the lipid-bilayer for surface area change is considered for both the whole RBC surface and for individual triangular element, as the lipid-bilayer is anchored to the cytoskeleton through transmembrane proteins and therefore, the movement of lipid molecules over the membrane is restricted [55]. The first part of Eq (12) represents the total surface area constraint whereas the second part of Eq (12) represents the triangular element surface area constraint.

A BCM-based membrane bending energy approach is implemented in the CG-RBC membrane model to predict SDE transformations. Therefore, in addition to above energy components, another two constraints are included to the system to maintain a reference area-difference between bilayer-leaflets and to maintain a reference total-membrane-curvature. The area-difference between bilayer-leaflets with $D_{0}$ monolayer thickness can be estimated based on a discrete approximation as follows [19, 49]:

$$\Delta A=2\;D_{0}\;\sum_{j\;\epsilon \;1\ldots \;N_{s}}M_{j}$$ (14)

The energy penalty of the membrane due to the constraint to maintain reference bilayer-leaflet-area-difference $\left(E_{Area-difference}\right)$ is considered to be proportional to the square of the difference between ΔA and the reference bilayer-leaflet-area-difference $\left(\Delta A_{0}\right)$, and can be given as follows:

$$E_{Area-difference}=\;\frac{1}{2}\;\frac{\pi \;k_{ad}}{D_{o}^{2}}\;{\left(\frac{\Delta A-\;\Delta A_{o}}{A}\right)}^{2}\;A$$ (15)

where, $k_{ad}$ is the bilayer-leaflet-area-difference constraint coefficient. Following-up on the discussion in section; ‘Computational simulations of RBC’, ADE model converges to BCM at $\bar{\kappa }/\kappa \;\to \;\infty$, and therefore, it is reasonable for $k_{ad}$ to adopt a significantly higher value than κ. Furthermore, the comparison between Eqs (4) and (15) shows that $\bar{\kappa }\;\approx \;4.0\;k_{ad}$. In the ADE model, κ and $\bar{\kappa }$ are in the same order [19, 49], and therefore the competition between spontaneous membrane curvature and bilayer-leaflet-area-difference, leads to consistent RBC shapes as minimum energy configuration for specified reference conditions. However, for the BCM model, inconsistent RBC shapes can arise at similar $\Delta A_{0}$ as $\bar{\kappa }$ is of higher order than κ, and therefore is the dominant component. ΔA is the integrated mean curvature over the membrane surface, and therefore $\Delta A_{0}$ can be achieved at multitude combinations of direction-dependent mean curvature at any point on the membrane surface. Equivalently, in the case of present discrete approximation of ΔA, the reference $\Delta A_{0}$ can be achieved at multitude convex and concave combinations of triangular-pair arrangement over the membrane surface. Therefore, to restrict the inconsistency of resultant RBC shape, the total-membrane-curvature constraint is introduced to the system. The total-membrane-curvature can be presented as the integrated direction-independent mean curvature over the membrane surface. Consequently, the minimum energy configuration of a RBC at specified $\Delta A_{0}$ can be obtained at a consistent convex and concave combination of triangular-pair arrangement over the membrane surface, which is also in agreement with the specified reference total-membrane-curvature $\left(C_{0}\right)$. The instantaneous total-membrane-curvature (C) for any RBC shape is higher than ΔA since it considers the absolute value of mean curvature over the membrane surface. C can be estimated as follows:

$$C=\;2\;D_{o}\;\sum_{j\;\in 1\ldots N_{s}}\left|M_{j}\right|$$ (16)

Therefore, the energy penalty of the membrane arising due to the constraint to maintain specified $C_{0}$ $\left(E_{Total-curvature}\right)$ is defined as follows:

$$E_{Total-curvature}=\;\frac{1}{2}\;\frac{\pi \;k_{C}}{D_{o}^{2}}\;{\left(\frac{C-\;C_{o}}{A}\right)}^{2}\;A$$ (17)

where $k_{C}$ is the total-membrane-curvature constraint coefficient, and one can expect $k_{C}$ to be within the same order of $\bar{\kappa }$. The total free energy of the membrane (E), can therefore be determined by the summation of all these energy components, such that:

$$E=\;E_{Stretching}+\;E_{Bending}+\;E_{Surface\;Area}+\;E_{Volume}+\;E_{Area-difference}+\;E_{Total-curvature}$$ (18)

It is assumed that the membrane particles move over the RBC membrane to achieve the minimum free energy state. The force $\left(F_{i}\right)$ acting on the $i^{th}$ membrane vertex point at the point $r_{i}$ on the surface can be derived from the principle of virtual work, such that,

$$F_{i}=\;-\;\frac{\partial \;E}{\partial \;r_{i}},\;i\;\in 1\ldots \;N_{v}$$ (19)

The motion of $i^{th}$ point can be described from the Newton’s second law of motion as follows:

$$F_{i}=\;m_{i}\;{\ddot{r}}_{i}+\;c\;{\dot{r}}_{i}$$ (20)

where, $m_{i}$ is the mass of $i^{th}$ particle, dot (.) is the time derivative and c is the viscosity of RBC membrane.

### Model parameters for RBC shape prediction

The initial spherical geometry is built up on an icosahedron of equilateral triangular faces that is inscribed within a sphere [19, 49, 56] of radius $R_{RBC}.\;R_{RBC}$ is determined for a sphere having equivalent surface area to a RBC. The physiological RBC surface area being ~ 140 μm^2^ [2, 5], $A_{0}$ is selected as 140 μm^2^ and therefore the estimated $R_{RBC}\;$= 3.34 μm. The refinement of triangulation is obtained by generating new vertices at midpoints of each triangular face edge, and combining the vertices together such that the preceding triangular surface is divided into four smaller triangles. The new vertices are then projected radially onto the spherical surface with the radius $R_{RBC}$. The minimum required particle resolution is determined such that the percentage error (ɛ) for estimating $E_{Stretching},\;E_{Bending},\;A,\;V$ and ΔA for the initial spherical geometry falls below 1.0% against the analytical estimations. The present CG-RBC membrane model consists of $N_{V}$ = 2,562 membrane vertex points, $N_{t}$ = 5,120 triangular elements and $N_{S}$ = 7,680 adjacent particle-particle connections. The quality of the triangulation is determined by the distribution of adjacent particle-particle connection length and the distribution of adjacent particle-particle connection for each vertex point [50]. The former is attributed to the ratio of standard deviation of the adjacent particle-particle connection length to the average adjacent particle-particle connection length. A smaller value is preferred for this ratio, and for the present consideration the value of this ratio is only 0.065. It is also preferred to have a higher percentage of vertex points having 6 adjacent particle-particle connections. In the present CG-RBC membrane, only the 12 vertices (= 0.47%) on the initial icosahedron have 5 particle-particle connections, whereas all the remaining membrane vertex points (= 99.53%) have 6 particle-particle connections. Therefore, the CG-RBC membrane has high quality of triangulation, and the effects of inhomogeneity can be considered minimal during SDE transformation.

The reference state of the cytoskeleton is assumed as an ellipsoid at 0.94 of volume of a sphere having $A_{0}$ surface area, and the equilibrium length of the spectrin link $l_{0}$ is determined at this reference state. The membrane shear modulus, ${µ}_{0}$ is set at 4.0 μNm^-1^ and agrees with the physiological shear modulus [9, 50], whereas the parameters $k_{p},\;p$ and $l_{max}$ are estimated at the room temperature, T = 296.15 K, and at m = 2 and $x_{0}$ = 0.45 [50].

The experimentally estimated RBC membrane bending modulus lies in the range of 1 x 10^−19^–7 x 10^−19^ Nm [9, 57], and therefore κ is selected to be 2.5 x 10^−19^ Nm. The constraint coefficients $k_{A},\;k_{a},\;k_{v},\;k_{ad}$ and $k_{C}$ are set to 1 x 10^−3^ Nm^-1^, 5 x 10^−5^ Nm^-1^, 100 Nm^-2^, 7.5 x 10^−17^ Nm and 2.5 x 10^−17^ Nm, respectively to achieve reference constraint conditions. The reference triangular element surface area $\left(A_{k,0}\right)$ is set at the corresponding triangular element area at cytoskeletal reference state. The reference cell volume $\left(V_{0}\right)$ is considered as 93.48 μm^3^ in agreement with the physiological RBC volume [2, 5], and is 0.6 of volume of the sphere of radius $R_{RBC}$. The lipid monolayer thickness $\left(D_{0}\right)$ is considered as 2 nm. $\Delta A_{0}/A_{0}$ and $C_{0}/A_{0}$ are set within the ranges of 0.05% - 0.30% and 0.30% - 0.90% respectively to predict different stages of SDE transformation.

The motion of RBC membrane particles to reach the equilibrium state, is estimated at c = 1 x 10^−7^ Nsm^-1^ and $m_{i}$ = 1 x 10^−9^ kg. The parameters c and $m_{i}$ do not affect the equilibrium RBC shape as they only control the speed of achieving the equilibrium state [54]. The updated velocity (${\dot{r}}_{i}$) and the position ($r_{i}$) of the $i^{th}$ vertex at time (t+Δt) from time (t) is given as follows;

$${\dot{r}}_{i}\left(t\;+\;\Delta t\right)\;=\;c\;{\dot{r}}_{i}\left(t\right)\;+\;{\ddot{r}}_{i}\left(t\right)\;\Delta t$$ (21)

$$r_{i}\left(t\;+\;\Delta t\right)\;=\;r_{i}\left(t\right)\;+\;{\dot{r}}_{i}\left(t\;+\;\Delta t\right)\;\Delta t$$ (22)

where, Δt is the time-step for succeeding iteration. The iterations are continued until the RBC membrane reaches the equilibrium state, which is the minimum free energy state of the RBC membrane at given reference conditions. In the present computational implementation of CG-RBC membrane model, the equilibrium cell state is acknowledged when the change between each analogous energy component ($E_{Stretching},\;E_{Bending},\;E_{Surface\;Area},\;E_{Volume},\;E_{Area-difference},\;E_{Total-curvature}$) at two successive iterations, is less than 1 x 10^−7^ in the order of energy component in consideration.

### Validation of CG-RBC membrane model

#### Qualitative validation of CG-RBC membrane model predicted SDE transformation

RBC SDE shape transformation is predicted at reference reduced cell volume (ν) and fitting combinations of $\Delta A_{0}$ and $C_{0}$ conditions. ν specifies the cell volume relative to the sphere having equivalent cell surface area, and is defined as follows:

$$\nu =\;\frac{V_{0}}{4/3\;\pi \;R_{RBC}^{3}}$$ (23)

**Fig 3:**
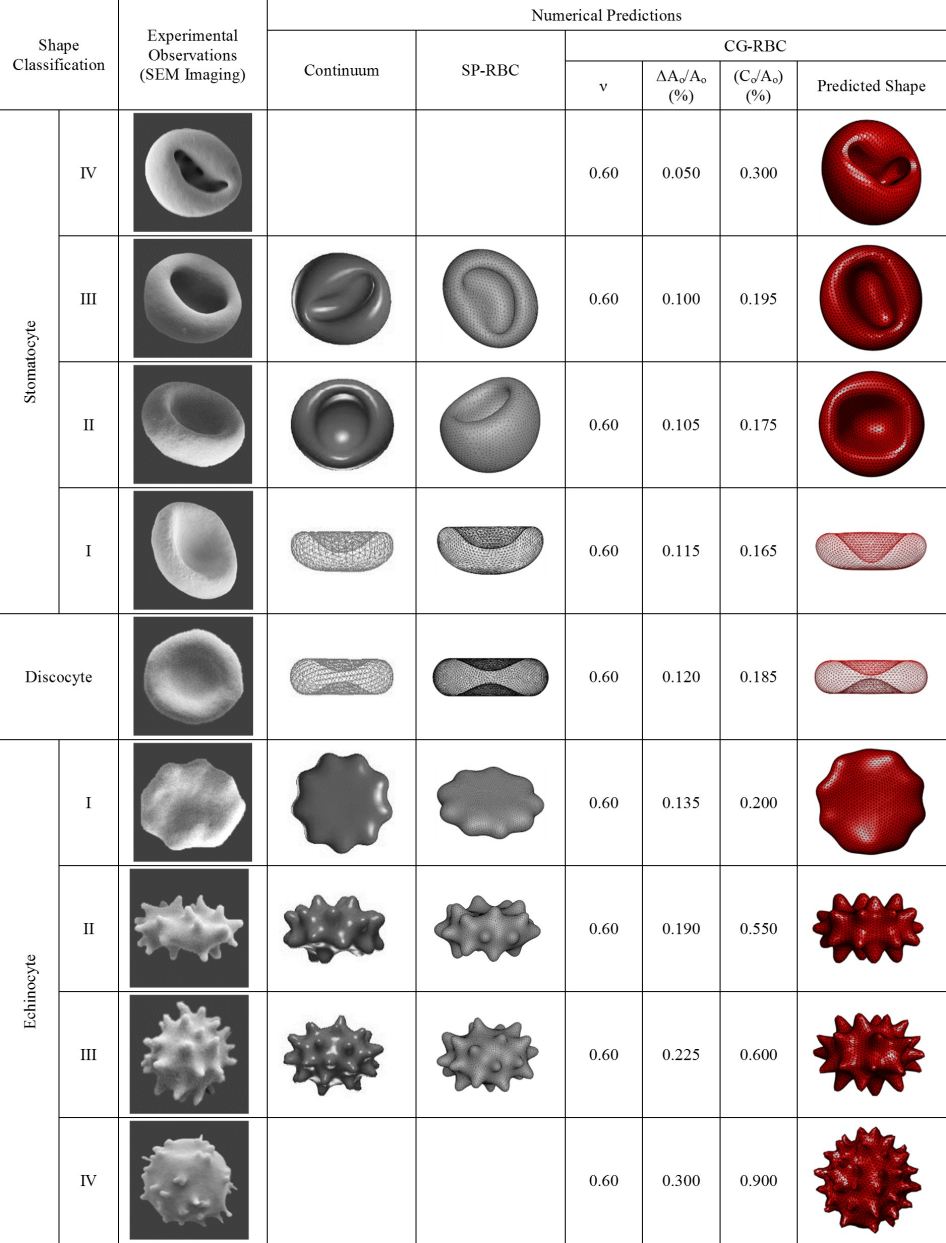
Comparison of SDE shape transformation predicted using CG-RBC membrane model with scanning electron microscopy (SEM) experimental observations and different numerical prediction techniques. The corresponding CG-RBC membrane model parameters of reference reduced cell volume (ν), bilayer-leaflet-area-difference $\left(\Delta A_{0}\right)$ and total-membrane-curvature $\left(C_{0}\right)$ are indicated next to the predicted RBC shape. Representative images for the shapes predicted by Lim et al. [19, 28, 49] through continuum approach (G. H. W. Lim, personal communication, March 19, 2019), and by Chen and Boyle [15] through SP-RBC model ((M. Chen, personal communication, September 13, 2018)) are presented. These images are similar but not identical to the original images from Lim et al. [19, 28, 49] and Chen and Boyle [15], and therefore for illustrative purposes only.

Several shape classes can be observed as the minimum energy configuration at combinations of different ν,ΔAo and $C_{0}$. The SDE shape transformation presented in Fig 3 is predicted at ν= 0.6, 0.05% $\leq \;\Delta A_{0}/A_{0}\;\leq$ 0.30% and 0.30% $\leq \;C_{0}/A_{0}\;\leq$ 0.90%, and is compared with both experimental observations and different numerical predictions. Experimental observations from SEM imaging (refer to S1 Appendix for detailed information. The laboratory protocol is available at http://dx.doi.org/10.17504/protocols.io.yvhfw36), and the numerical predictions by Lim et al. [19, 28, 49] and Chen and Boyle [15] are used for the comparison in Fig 3. It can be observed that the predicted SDE transformation through the implemented CG-RBC membrane model agrees well with the experimentally observed and numerically predicted results. The implemented CG-RBC model is also capable of predicting the sphero-stomatocyte (stomatocyte IV) and sphero-echinocyte (echinocyte IV) stages as stable minimum energy configurations, which have not been predicted by other numerical approaches. The predicted sphero-stomatocyte shape consists with small interior buds that are attached to the membrane surface whereas the sphero-echinocyte shape consists of small sharp projections as expected. Therefore, the CG-RBC membrane model can predict the complete SDE transformation at constant cell surface area and cell volume constraints.

#### Quantitative validation of CG-RBC membrane model predicted SDE transformation

In addition to the qualitative validation presented in Fig 3, a quantitative analysis is performed on the predicted RBC shapes obtained using the CG-RBC membrane model against experimental data extracted from 3D confocal microscopy imaging of RBC. Different shapes were obtained by changing buffer composition before labelling and fixing the cells (refer to S2 Appendix for detailed information. The laboratory protocol is available at http://dx.doi.org/10.17504/protocols.io.yjyfupw). Imaging data were then analysed using numerical methods to recreate a triangulated mesh over the surface of the cells. Experimental measurements were calibrated using fluorescent beads with a known diameter and imaged at the same time as the RBC. Calibration enabled accurate measurements of RBC surface area and volume.

**Fig 4:**
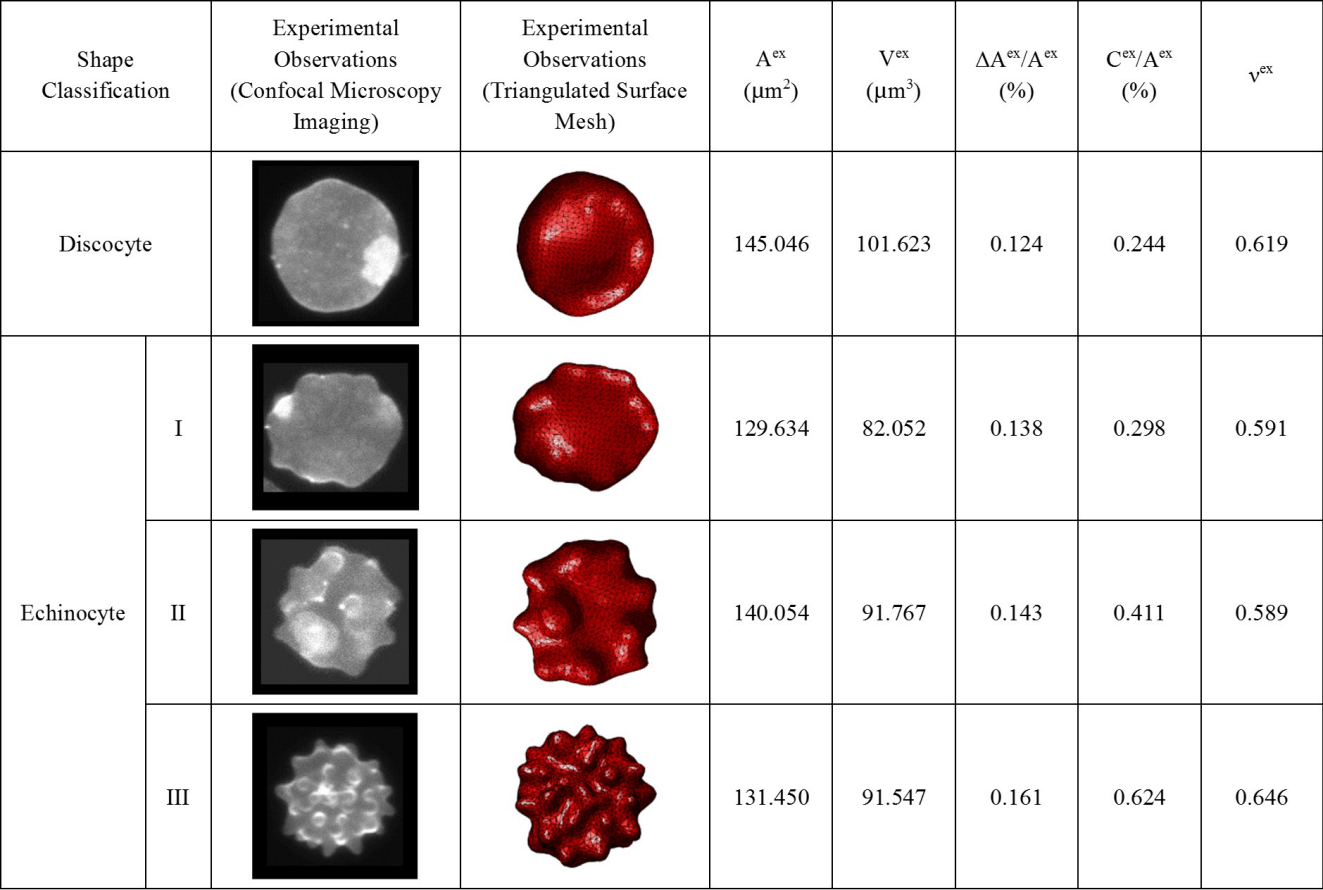
Summary of estimated cell surface area $\left(A^{ex}\right)$, cell volume $\left(V^{ex}\right)$, bilayer-leaflet-area-difference $\left(\Delta A^{ex}\right)$, total-membrane-curvature $\left(C^{ex}\right)$ and equivalent reduced cell volume $\left(\nu^{ex}\right)$ for the experimentally observed discocyte, echinocyte I, echinocyte II and echinocyte III RBC shapes.

The cell surface area $\left(A^{ex}\right)$, cell volume $\left(V^{ex}\right)$, bilayer-leaflet-area-difference $\left(\Delta A^{ex}\right)$ and total-membrane-curvature $\left(C^{ex}\right)$ are estimated from these generated triangulated surfaces for four randomly selected RBC having discocyte, echinocyte I, echinocyte II and echinocyte III shapes, and are summarized in Fig 4. The estimated $\Delta A^{ex}/A^{ex}$ and $C^{ex}/A^{ex}$ for these experimentally obtained RBC are introduced to the CG-RBC membrane model as reference $\Delta A_{0}/A_{0}$ and $C_{0}/A_{0}$ parameters, and the resultant shape is determined at equivalent reduced cell volume $\left(\nu^{ex}\right)$ constraint. $\nu^{ex}$ for the randomly selected RBC each having discocyte, echinocyte I, echinocyte II and echinocyte III shapes, is defined according to Eq (24).

$$\nu^{\text{ex}}=\;\frac{V^{ex}}{Volume\;of\;a\;sphere\;having\;equivalent\;A^{ex}}$$ (24)

Assuming rigid body conditions, the centre of mass of the whole cell and its three-principle axes of inertia are determined for each experimentally observed and numerically predicted cell shapes. $H_{1},\;H_{2}$ and $H_{3}$ are defined as the distance between the furthest vertex points on RBC membrane surface along these three-principle axis of inertias of the cell such that $H_{1}\;\leq \;H_{2}\;\leq \;H_{3}$, and used to estimate the cellular measurements: the normalized cell length $\left(H_{x}\right)$, normalized cell thickness $\left(H_{z}\right)$ and shape factor (SF). $H_{x}$ is defined as the ratio between $H_{3}$ and the equivalent spherical radius $\left(R^{*}\right)$, where $R^{*}$ is the radius of the sphere having equivalent cell surface area. Similarly, $H_{z}$ is defined as the ratio between $H_{1}$ and $R^{*}.\;SF\;=\;H_{1}\;/\sqrt{H_{2}\;\times \;H_{3}}$ [58], and indicates the sphericity of the cell. The cell becomes more and more spherical as SF reaches the value 1, and becomes more and more flattened disc as SF reaches 0.

**Fig 5:**
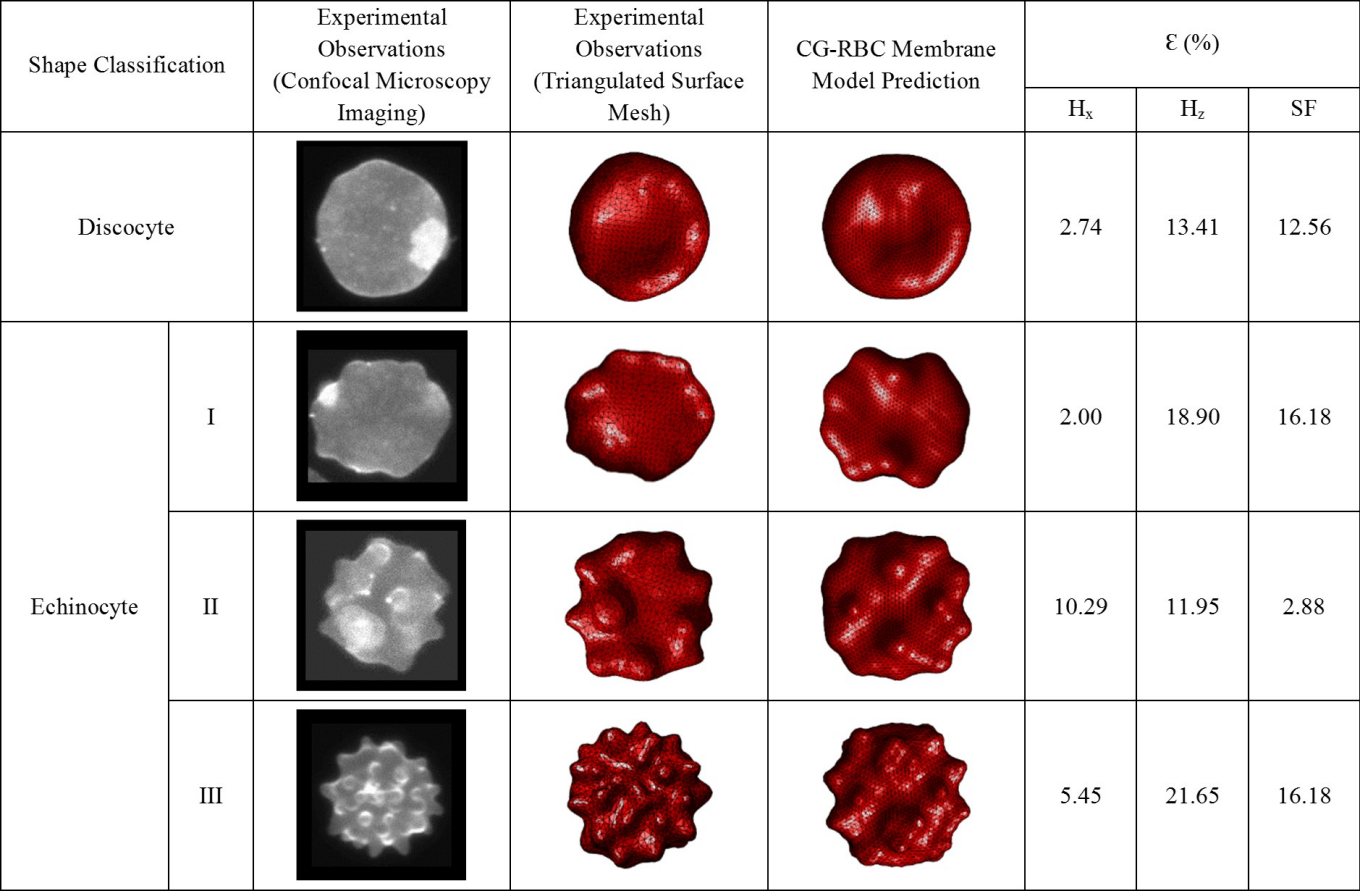
Summary of estimated percentage error (ɛ) for normalized cell length $\left(H_{x}\right)$, normalized cell thickness $\left(H_{z}\right)$ and shape factor (SF) between corresponding experimentally observed and CG-RBC membrane model predicted RBC shapes.

These three cellular measurements are used to quantitatively compare the corresponding experimentally observed and numerically predicted RBC shapes. The estimated percentage error values (ɛ) for $H_{x},\;H_{z}$ and SF between corresponding experimentally observed and numerically predicted RBC shapes are presented in Fig 5. It can be observed that the resulting shapes obtained through CG-RBC membrane model agree well with the shapes derived from 3D confocal microscopy imaging. The values of ɛ for $H_{x},\;H_{z}$ and SF are reasonable and the maximum ɛ (= 21.65%) is for the $H_{z}$ in the case of echinocyte III RBC shape. However, any experiment error during 3D confocal microscopy imaging and image analysis can lead to ɛ while triangulated surface generation can also be another contributing factor. Therefore, the resulting ɛ is the resultant effect of any experimental errors, any triangulated surface generating errors and any error in CG-RBC shape predictions. It can be observed that ɛ is generally higher for $H_{z}$ than $H_{x}$, which contributes to ɛ for SF as well. The experimentally observed cell being not completely lying on the bottom surface of the imaging chamber leads to over prediction of $H_{z}$. Therefore, better fixation of the cell during 3D confocal imaging experiments can improve the accuracy of $H_{z}$ measurements. In addition, the accuracy of $\Delta A^{ex}/A^{ex}$ and $C^{ex}/A^{ex}$ estimations can be improved by generating a more homogeneous triangular network having similar triangular edge lengths. By this means, it is possible to improve the accuracy of experimentally derived measurements, which can improve the comparability between analogous experimental observations and numerical predictions. However, the maximum ɛ being 21.65%, is acceptable based on all these uncertainties, and therefore the CG-RBC model is capable of quantitatively represent different SDE morphologies.

## Results and discussion

### Effect of RBC reduced volume on SDE transformation

**Fig 6:**
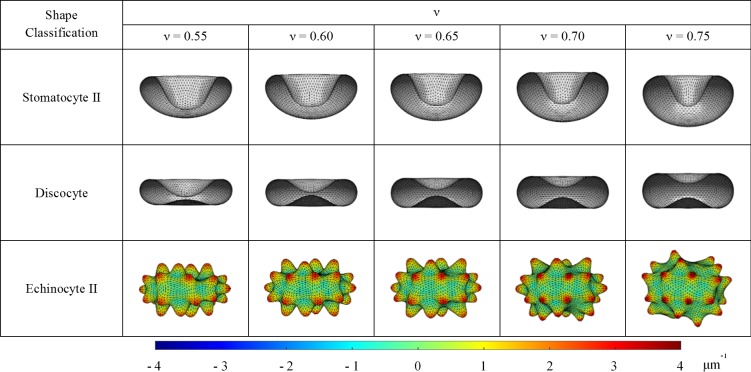
CG-RBC membrane model predicted stomatocyte II, discocyte and echinocyte II shapes at reduced cell volumes of ν = 0.55, 0.60, 0.65, 0.70 and 0.75. The coloured regions of the echinocyte II shape represent the membrane curvature on its’ vertex points $\left(C_{V,i}\right)$.

The CG-RBC membrane model is used to investigate the effect of reduced cell volume (ν) on SDE transformation. In an isotonic environment, ν is equivalent to 0.6 whereas ν increases or decreases in hypotonic and hypertonic environments, respectively. The insight on SDE behaviour at different tonicity conditions facilitate the better understanding of SDE behaviour at specific shape-transforming conditions. Therefore, RBC shapes are predicted at similar reference conditions of $\Delta A_{0}$ and $C_{0}$ as presented in Fig 3 and at different ν condition (ν = 0.55, 0.60, 0.65, 0.70 and 0.75). The stable minimum energy configurations at specified $\Delta A_{0},\;C_{0}$ and ν are illustrated in Fig 6 for stomatocyte II, discocyte and echinocyte II stages. The BCM model can predict stable vesicle shapes if the ratio of $\bar{\kappa }/\kappa$ is above the critical value at ν [23, 48], and for the present consideration, $\bar{\kappa }/\kappa$ = 150, a value well above the critical ratio of $\bar{\kappa }/\kappa$ = (0, 20) for ν = [0.55, 0.75].

It can be observed that the predicted RBC shapes become more spherical as expected with the increase in $\nu .\;H_{x}$ for the SDE transformation gradually reduces whereas $H_{z}$ increases, and therefore the SF also increases gradually with the increase in ν. The membrane curvature corresponding to $i^{th}$ membrane vertex point $\left(C_{V,i}\right)$ is defined as follows:

$${\text{C}}_{\text{V},\;\text{i}}=\;\sum_{\text{jj}\;\in 1\;\ldots {\;\text{N}}_{\text{s},\;\text{i}}}\frac{{\text{M}}_{\text{jj}}}{\Delta {\text{A}}_{\text{jj}}}$$ (25)

where, $N_{S,i}$ is the number of associated spectrin links with the $i^{th}$ membrane vertex point, and $M_{jj}$ and $\Delta A_{jj}$ represent the membrane curvature at the triangle-pair that shares the ${jj}^{th}$ spectrin link and the surface area associated with it, respectively. It can also be observed (refer to Fig 6) that the maximum $C_{V,i}$ for echinocyte II, echinocyte III and echinocyte IV shapes increase rapidly whereas the minimum $C_{V,i}$ for stomatocyte II, stomatocyte III and stomatocyte IV decrease with increase in ν. Therefore, it can be summarized that the absolute $C_{V,i}$ for stomatocyte II, III and IV stages and echinocyte II, III and IV stages increases rapidly with the increase in ν whereas no significant change can be observed for stomatocyte I, discocyte and echinocyte I shapes. In general, for the stomatocyte shape, the oval shaped concavity mouth becomes more circular and its minimum cell thickness increases with ν. Similarly, the predicted discocyte shape shows asymmetric. biconcavity at low ν, which becomes more symmetric with increasing ν. The minimum cell thickness of discocyte also increases with ν. For the echinocyte shapes, the spicules become narrower and sharper with uneven distribution over the membrane surface with the increase of ν. Therefore, the outcomes of the study on the effect of ν on SDE transformation suggest that though the predicted SDE shape class has not been affected by the changes in ν, the features of the resultant RBC shape is affected. Therefore, the SDE transformations with equivalent reference conditions of $\Delta A_{0}$ and $C_{0}$ behave differently at different ν conditions, and one should consider all the relevant reference conditions to accurately predict the resultant RBC morphology.

### Effect of RBC elastic length scale on SDE transformation

It has been found that the RBC membrane bending modulus differs for different SDE morphology [59], whereas the RBC membrane stiffness increases with in-vitro cell ageing and several diseased conditions such as malaria infection, SCA, HS and HE [9]. Therefore, the CG-RBC membrane model is used to investigate the effect of elastic length scale $\left(\Lambda_{el}\right)$ on SDE transformation. $\Lambda_{el}$ is the relation of RBC membrane bending modulus (κ) to its shear modulus $\left({µ}_{0}\right)$, and is defined as follows [13]:

$$\Lambda_{el}=\;\sqrt{\frac{\kappa }{\mu_{0}}}$$ (26)

Li et al. [60] have suggested that the $\Lambda_{el}$ should be above a critical value (~ 0.37 μm) at the spherical cytoskeletal reference state in order to obtain the discocyte shape as the possible minimum free energy configuration. Otherwise, stomatocyte shape is resulted as the minimum free energy configuration. However, Mukhopadhyay et al. [13] have used $\Lambda_{el}$ = 0.28 μm at zero pre-stressed cytoskeletal reference state, whereas Lim et al. [19, 49] have used $\Lambda_{el}$ = 0.28 μm at the ellipsoidal cytoskeletal reference state, in order to investigate RBC shapes and shape transformations. Moreover, Tsubota [54] has investigated the critical value of $\Lambda_{el}$ at the shape transition between stomatocyte and discocyte for several forms of out-of-plane bending energy estimations. In the present study, SDE transformation presented in Fig 3 is obtained at $\Lambda_{el}$ = 0.25 μm at an ellipsoidal cytoskeletal reference state, and agrees with the critical limits of $\Lambda_{el}$ suggested by Tsubota [54] for the corresponding out-of-plane bending energy form.

**Fig 7:**
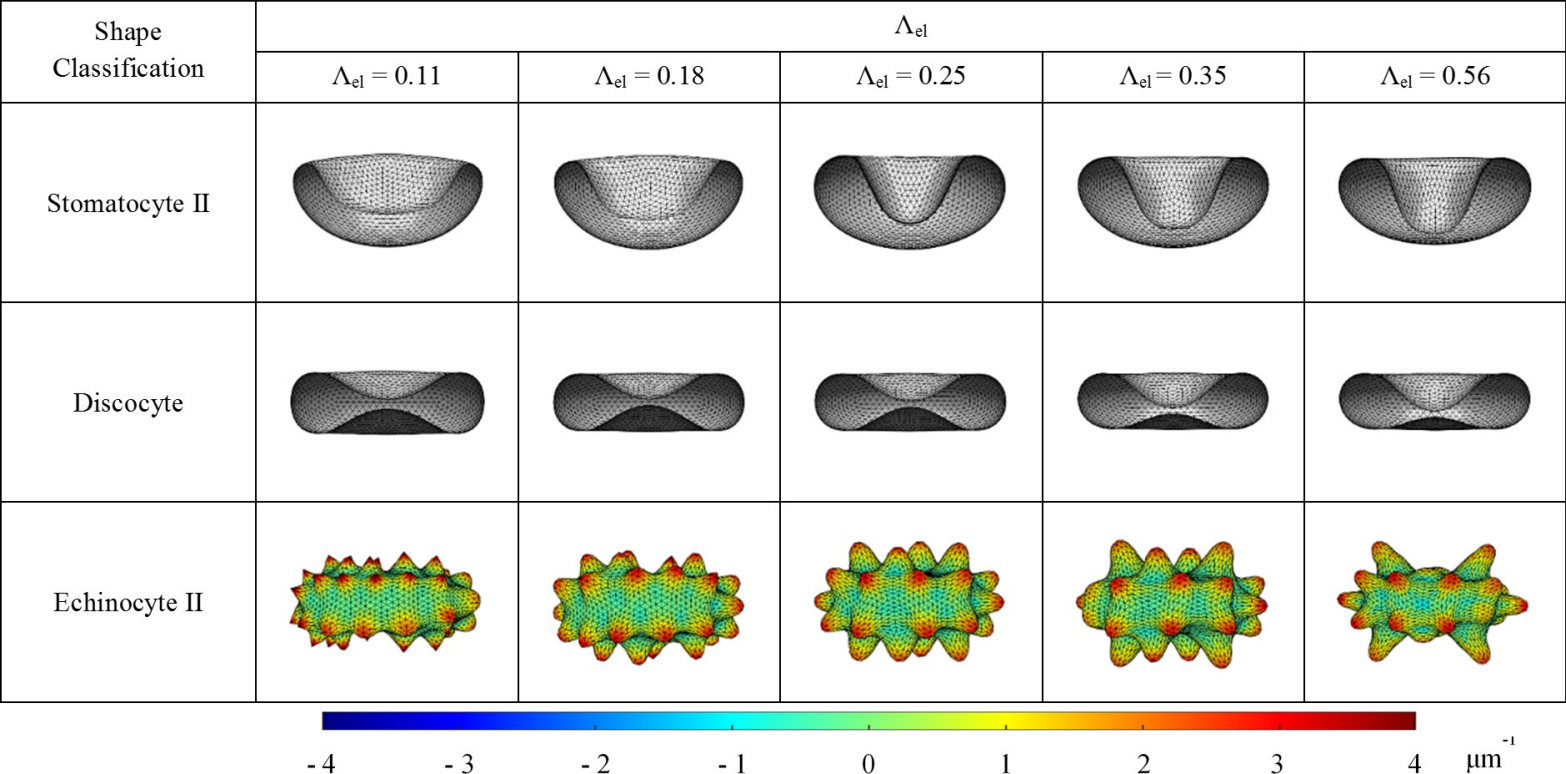
CG-RBC membrane model predicted stomatocyte II, discocyte and echinocyte II shapes at varying elastic length scale, $\Lambda_{el}$ = 0.11, 0.18, 0.25, 0.35 and 0.56. The coloured regions of the echinocyte II shape represent the membrane curvature on its’ vertex points $\left(C_{V,i}\right)$.

Several $\Lambda_{el}\;[\Lambda_{el}\;$(μm) = 0.11, 0.18, 0.25, 0.35 and 0.56] values are achieved at 5.0, 2.0, 1.0, 0.5 and 0.2 multiplications of ${µ}_{0}\;({µ}_{0}$ = 4.0 μNm^-1^) at constant κ(κ = 2.5 × 10^−19^ Nm), where ${µ}_{0}$ and κ are in the physiological range for cytoskeletal shear modulus and membrane bending modulus. RBC shapes are predicted at similar reference conditions of $\Delta A_{0},\;C_{0}$, and ν as presented in Fig 3 but at varying $\Lambda_{el}$. The behaviour of SDE transformation at varying $\Lambda_{el}$ is illustrated in Fig 7 for stomatocyte II, discocyte and echinocyte II stages, which each represents the stomatocyte, discocyte and echinocyte stages, respectively. The cellular measurements; $H_{x},\;H_{z}$ and SF do not show significant variation with the change in $\Lambda_{el}$. However, the maximum $C_{V,i}$ for echinocyte II, echinocyte III and echinocyte IV shapes decreases rapidly whereas the minimum $C_{V,i}$ for stomatocyte II, stomatocyte III and stomatocyte IV increases with the increase in $\Lambda_{el}$. Therefore, it can be summarized that the absolute $C_{V,i}$ for stomatocyte II, III and IV stages and echinocyte II, III and IV stages decreases rapidly with the increase in $\Lambda_{el}$ whereas no significant change can be observed for stomatocyte I, discocyte and echinocyte I shapes. In general, for the stomatocyte shape, the oval shaped concavity mouth becomes more circular and its minimum cell thickness decreases with $\Lambda_{el}$. Similarly, the predicted discocyte shape shows asymmetric biconcavity at high $\Lambda_{el}$ while the more symmetric biconcavity becomes less symmetric with increasing $\Lambda_{el}$. However, the minimum cell thickness of discocyte does not indicate significant change with $\Lambda_{el}$. For the echinocyte shapes, the spicules become broader and the spicule size become irregular with the increase of $\Lambda_{el}$. At lower $\Lambda_{el}$ values, the contribution from membrane bending energy component becomes less significant and therefore result in higher values of absolute $C_{V,i}$ for equilibrium RBC shapes. On the contrary, the membrane bending energy becomes more dominant at higher $\Lambda_{el}$ values and restricts $C_{V,i}$ for equilibrium RBC shapes. Therefore, smoother RBC membrane can be observed and it becomes difficult for equilibrium RBC shapes to reach the exact reference area-difference between bilayer-leaflets and total-membrane-surface-curvature conditions at higher $\Lambda_{el}$ values. These results reveal how the features of SDE transformation have been affected by the changes in $\Lambda_{el}$, and therefore provide valuable insight to SDE behaviour at different shape-transforming scenarios.

### Deformability of SDE transformation

**Fig 8:**
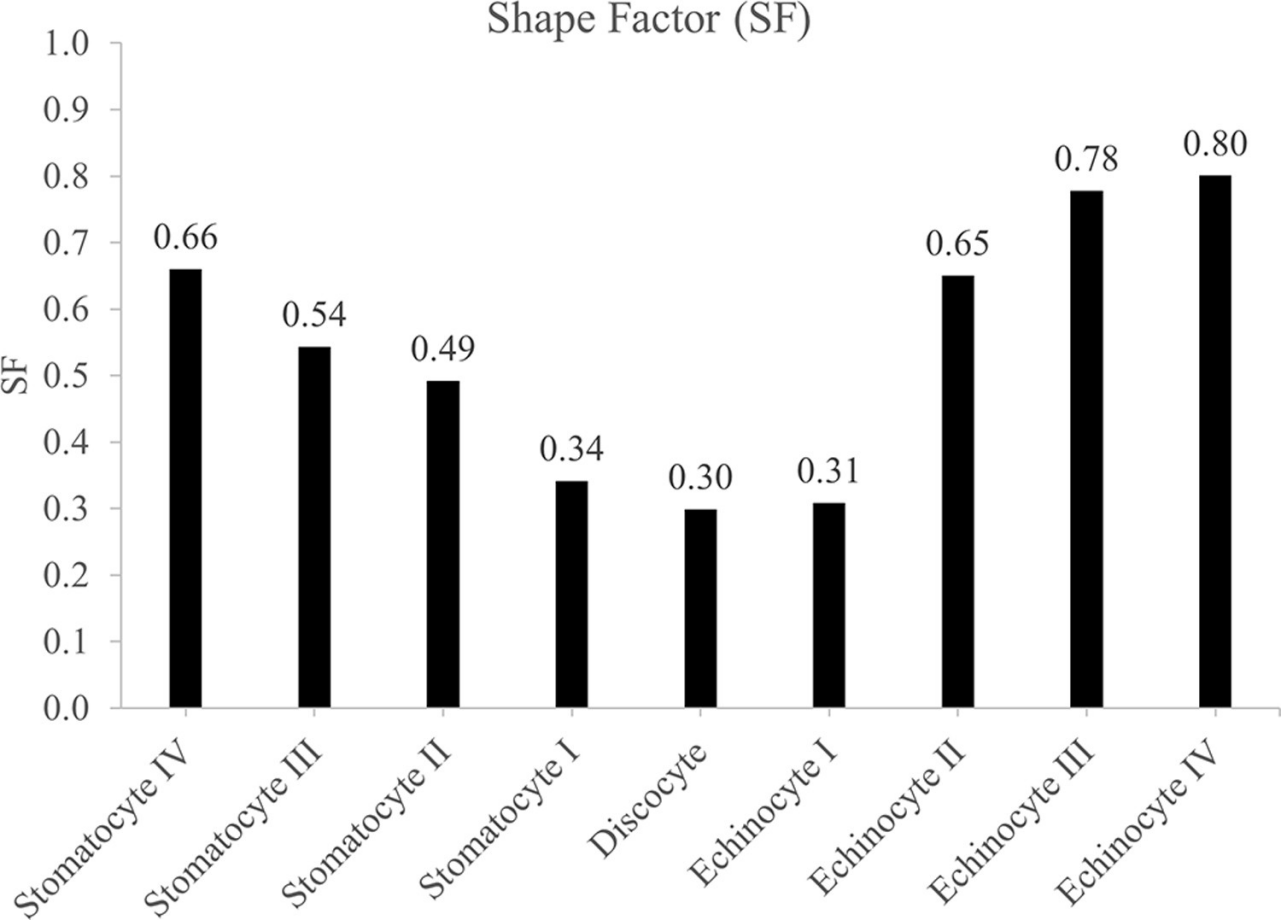
Shape Factor $\left(SF\right)$ for CG-RBC membrane model predicted stomatocyte IV—echinocyte IV RBC shapes.

The RBC membrane elasticity is a critical physiological index [61], and a RBC needs high elasticity to carry out its function as a gas carrier in the microcirculation. The main contributors of RBC deformability are cell geometry, intra-cellular and extra-cellular fluid viscosity and elastic properties of the cell membrane [62, 63]. Therefore, any change in cell deformability can be attributed to the change in one or more of these contributors. The highly deformable discocyte shape becomes more and more stiffer and finally rigid when it follows different stages through discocyte-spherocyte [64]. This phenomenon can be linked with the evolution of SF during SDE transformation, and Fig 8 summarises the estimated SF for the CG-RBC membrane model predicted SDE transformation stages that are presented in Fig 3. SF for the discocyte shape is the lowest, and increases progressively with stages I, II and II of stomatocytosis and echinocytosis where it reaches the maximum SF for sphero-stomatocyte and sphero-echinocyte shapes. The evolution of SF values can therefore be used as an indicator of RBC deformability during SDE transformation, and as such, the CG-RBC model has a strong potential in accurately predicting RBC deformability at different SDE stages.

Furthermore, the SDE transformation due to stomatocytic and echinocytic shape-transforming agents take place at constant membrane surface area and cell volume [11–16], and equivalently the CG-RBC membrane model predicted SDE transformation follows at constant reduced cell volume (ν = 0.6) (refer to Fig 3). Therefore, intra-cellular and extra-cellular fluid viscosity and/ or elastic properties of the cell membrane are the most likely deformability factors to be affected during reversible SDE transformation.

### Cytoskeletal reference state of RBC

The stress-free reference state of the cytoskeleton is a controversial subject [4, 42]. Several studies [4, 60] have suggested that a significantly lower cytoskeletal elasticity is needed to achieve a biconcave shape for a spherical cytoskeletal reference state. Otherwise, a non-spherical reference state; biconcave or ellipsoidal cytoskeletal reference states, must be assumed to achieve stable biconcave shape at physiological cytoskeletal elasticity. Lim et al. [19, 28, 49] numerically proposed that the cytoskeletal reference state of the RBC is most likely to be an ellipsoid with a reduced volume in the range of 0.925–0.976 of a sphere having equivalent surface area, and Khairy and Howard [31] have also suggested that the cytoskeletal reference state is most likely to be an ellipsoidal shape.

The investigation for the cytoskeletal reference state is carried out also with the implemented CG-RBC membrane model. The equilibrium spectrin length, $l_{0}$ is extracted at several cytoskeletal reference states, and used in CG-RBC membrane model to predict discocyte, echinocyte I, echinocyte II and echinocyte III shapes that are analogous to 3D confocal microscopy imaging observations (refer to section ‘Quantitative validation of CG-RBC membrane model predicted SDE transformation’). A similar approach as in Lim et al. [19, 49] is performed to generate cytoskeletal reference states and the CG-RBC membrane model is adopted to represent only the cytoskeletal spectrin network such that the stable minimum energy state is determined at set reference cytoskeletal surface area $\left(A_{0,\;Cyto}\right)$, cytoskeletal volume $\left(V_{0,Cyto}\right)$ and cytoskeletal reduced volume $\left(\nu_{Cyto}\right).\;A_{0,\;Cyto}$ is assumed to be equivalent to that of the RBC $\left(A_{0}\right)$ whereas the reference triangular element surface area of the cytoskeleton, $\left(A_{k,0,Cyto}\right)$ is set at the corresponding triangular element area at the initial spherical geometry having the radius R_RBC_. Several $V_{0,Cyto}$ conditions are studied such that $\nu_{Cyto}$ = 1.00, 0.99, 0.94, 0.89, 0.83, 0.78, 0.73, 0.68 and 0.63. The presence of $E_{Bending}$, in the form of a stronger bending modulus weakens the contribution from shear modulus and leads to an unstressed cytoskeletal state. In addition, the presence of cytoskeletal shear modulus though in weaker form avoids any numerical inconsistency. Therefore, a significantly higher bending modulus (κ= 5.0 × 10^−18^ Nm) is used at the physiological cytoskeletal shear modulus $({µ}_{0}\;$= 4.0 μNm^-1^) in order to predict the resultant cytoskeletal equilibrium state. The constraint coefficients; $k_{A},\;k_{a}$ and $k_{V}$ are set to 1 x 10^−3^ Nm^-1^, 5 x 10^−5^ Nm^-1^ and 100 Nm^-2^, respectively. The influence of any bilayer-leaflet-area-difference and total-membrane-curvature is neglected for cytoskeletal reference state generation. The stress-free equilibrium cytoskeletal state is acknowledged at the minimum free energy state, and corresponding $l_{0}$ is extracted at set $\nu_{Cyto}$. Next, discocyte, echinocyte I, echinocyte II and echinocyte III predictions described in the section; ‘Quantitative validation of CG-RBC membrane model predicted SDE transformation’, are repeated at estimated $l_{0}$ values.

**Fig 9:**
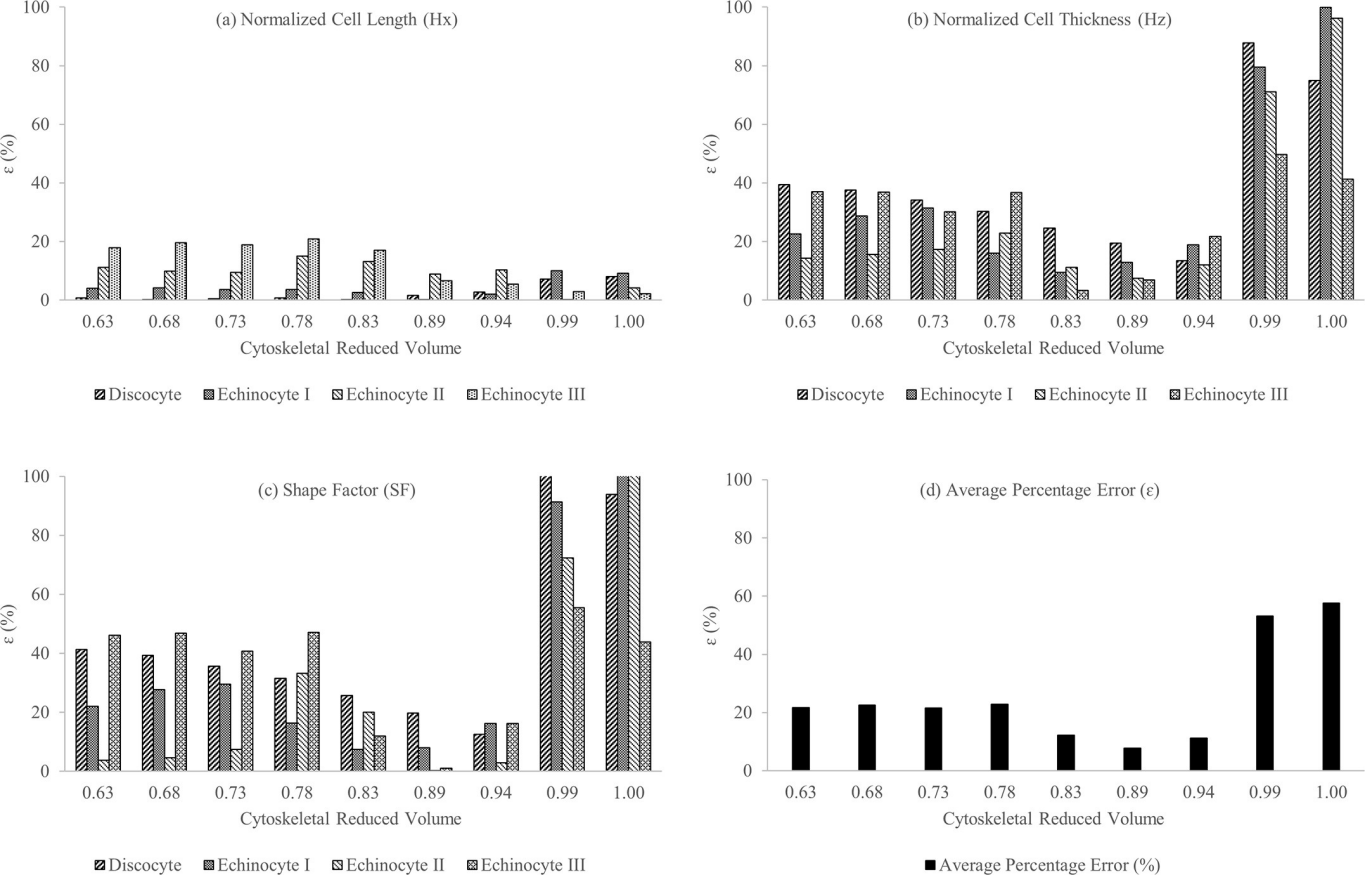
The percentage error (ε) for (a) normalized cell length $\left(H_{x}\right)$, (b) normalized cell thickness $\left(H_{z}\right)$, (c) shape factor (SF) and (d) resultant average error between corresponding experimentally observed and CG-RBC predicted discocyte, echinocyte I, echinocyte II and echinocyte III shapes at cytoskeletal reference states of $\nu_{Cyto}$ = 1.00, 0.99, 0.94, 0.89, 0.83, 0.78, 0.73, 0.68 and 0.63.

The error (ɛ) is once again estimated for the cellular measurements of $H_{x},\;H_{z}$ and SF between corresponding experimentally observed and CG-RBC predicted discocyte, echinocyte I, echinocyte II and echinocyte III shapes at stated cytoskeletal reference states. It can be observed (refer to Fig 9) that ɛ is very low for all $H_{x},\;H_{z}$ and SF cellular measurements at $\nu_{Cyto}\;$= 0.83,0.89 and 0.94 cytoskeletal reference states, whereas ɛ for $H_{x}$ and $H_{z}$ is significantly higher at $\nu_{Cyto}\;$= 1.00 and 0.99 cytoskeletal reference state conditions. The near spherical cytoskeletal reference state at $\nu_{Cyto}\;$= 1.00 and 0.99 results in higher cell thickness, which leads to the significantly higher ɛ at these cytoskeletal reference states.

**Fig 10:**
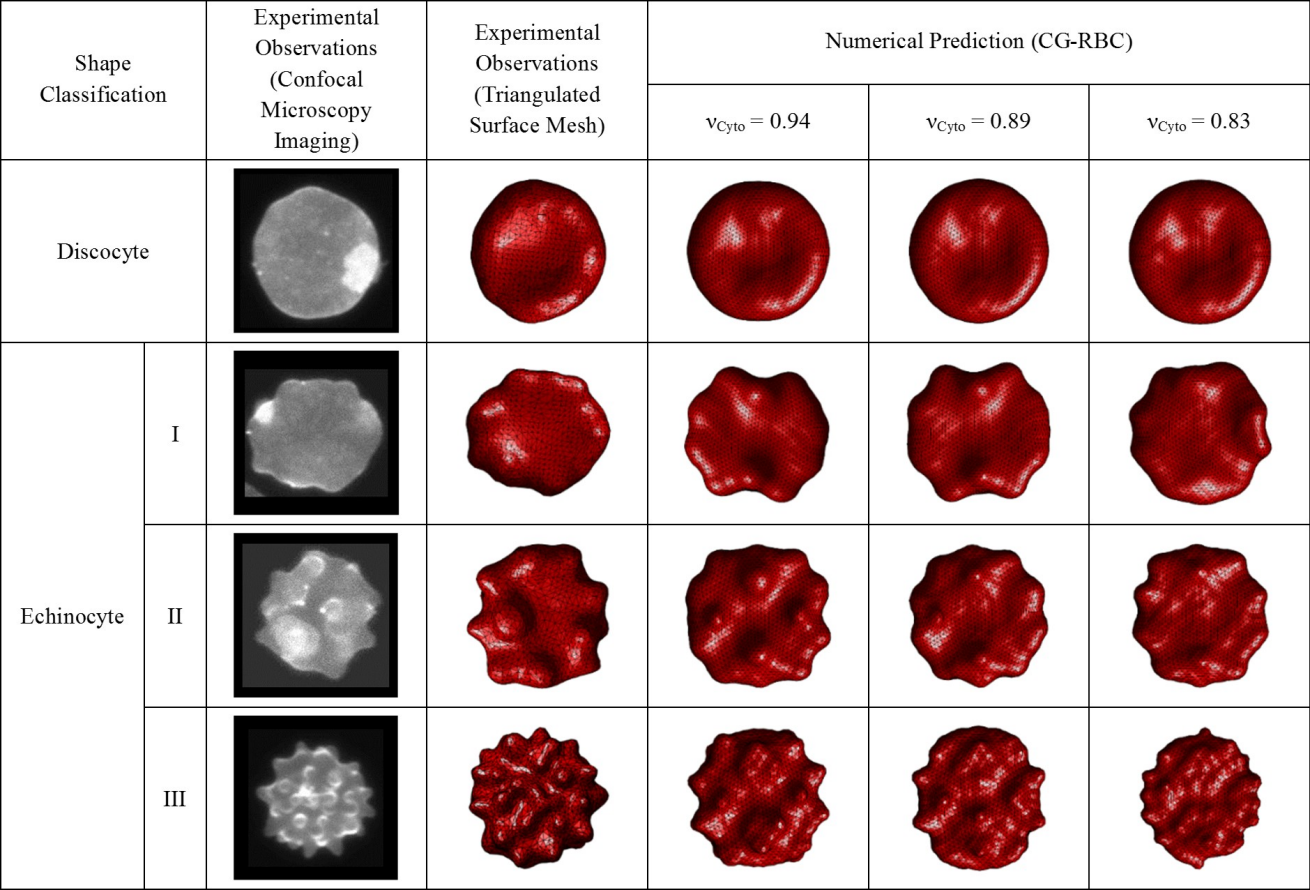
Qualitative comparison between corresponding experimentally observed and CG-RBC membrane model predicted discocyte, echinocyte I, echinocyte II and echinocyte III shapes at cytoskeletal reference states of $\nu_{Cyto}\;$= 1.00, 0.99, 0.94, 0.89, 0.83, 0.78, 0.73, 0.68 and 0.63.

The qualitative comparison (refer to Fig 10) of CG-RBC predicted discocyte, echinocyte I, echinocyte II and echinocyte III shapes at $\nu_{Cyto}\;$= 0.83, 0.89 and 0.94 cytoskeletal reference states, indicates that results at $\nu_{Cyto}$ = 0.94 cytoskeletal reference state agree well with the corresponding experimental observations. There is no significant difference between predicted discocyte shape at $\nu_{Cyto}$ = 0.83, 0.89 and 0.94. Although the echinocyte I shape at $\nu_{Cyto}$ = 0.83 agrees best with the experimental observation, echinocyte II and echinocyte III shapes at $\nu_{Cyto}$ = 0.83 do not agree well with the corresponding experimental observations. There is no significant difference between predicted echinocyte II shape at $\nu_{Cyto}\;$= 0.89 and 0.94, though the echinocyte III shape at $\nu_{Cyto}\;$= 0.94 has evenly distributed spicules over the membrane surface which agrees better with the experimental observations. Therefore, the most likely cytoskeletal reference state obtained from the CG-RBC membrane model is an ellipsoidal shape at around $\nu_{Cyto}$ = 0.94.

## Summary and outlook

A CG-RBC membrane model was developed in this study to improve the understanding of the stomatocyte-discocyte-echinocyte (SDE) transformation of RBC. In contrast to the existing ADE-based numerical simulation methodologies, a bilayer coupling model (BCM)-based membrane bending energy was employed in combination with a new additional constraint: the total-membrane-surface-curvature, in order to predict each stage of stomatocyte IV–echinocyte IV RBC shapes.

The CG-RBC membrane model produced similar RBC shapes with comparison to the existing numerically predicted SDE transformation that were based on either continuum or particle-based approaches. In addition to the commonly used qualitative validation of SDE transformation against experimental observations, a quantitative analysis is conducted in details to evaluate the accuracy and reliability of the shape predictions by the CG-RBC membrane model. Both qualitative and quantitative analyses of the CG-RBC membrane model predicted shapes against the experimentally observed shapes have proved that the newly developed CG-RBC membrane model can predict equivalent RBC shapes with reasonable accuracy. The membrane model was then employed to investigate the effect of cell reduced volume (ν) and elastic length scale $\left(\Lambda_{el}\right)$. Furthermore, the CG-RBC membrane model was successfully employed to identify the suitability of shape factor (SF) to estimate the deformability of each stage of SDE transformation. The predicted RBC shapes confirmed that the cytoskeletal reference state of the RBC is most likely an ellipsoidal shape, and is agreeable with the numerical predictions by Lim et al. [19, 28, 49] and Khairy and Howard [31] as well.

The CG-RBC membrane model is a general framework to predict the SDE transformation of RBC, and employs a coarse-grained approach to reduce the excessive computational cost for detailed description of the membrane structure. However, the model can be easily refined to represent the detailed RBC membrane structure and any molecular level changes in the presence of shape-transforming agents. The CG-RBC membrane model can be extended to predict the membrane budding and their detachment from RBC to form spherocyte; the RBC shape at higher concentrations of shape-transforming agents or at latter stages of in-vitro RBC storage. Similarly, it is possible to extend the current model to predict the RBC behaviour at defective cytoskeletal conditions; i.e. HE conditions. The equilibrium length of the spectrin links can be set individually, whereas the disruption of vertical connections between lipid-bilayer and cytoskeletal actin junctions can be incorporated into the model by cancelling $E_{Stretching}$ for the spectrin links attached to these actin junctions. The loss of membrane surface under HS conditions is possible to be introduced to the model by appropriate adjustments to ν. However, the cytoskeletal is under compression as it is attached to a lipid-bilayer having a lower surface area than a healthy cell. Therefore, the cytoskeletal reference state would need adjustments to discuss HS cell behaviour though current CG-RBC membrane model.

In addition, extending the present CG-RBC membrane model to discuss distinct RBC shape-transforming conditions, is a potentially interesting future research avenue. Each shape-transforming agent affects the bilayer-leaflets-area-difference in a unique manner and lead to different stages of SDE transformation. However, the process of changing the area-difference between bilayer-leaflets is unique for each shape-transforming agent. The present CG-RBC membrane model can be extended to incorporate equivalent model parameters at distinct shape-transforming conditions; disease conditions such as HS and HE; occurrence of echinocytic and stomatocytic shape-transforming agents; and RBC aging during in-vitro storage. Furthermore, the proposed CG-RBC membrane model can be easily adapted to consider other hypotheses such as Band 3 conformation [17] and cytoskeleton-bilayer interactions [9] induced RBC shape transformations.

The introduced quantitative analysis framework between experimentally observed and CG-RBC membrane model predicted RBC shapes, forms a feasible approach to better incorporate the biochemical effects into the model. Therefore, the presented CG-RBC membrane model facilitate better understanding of RBC behaviour at different shape-transforming conditions.

## Supporting information

S1 Appendix Methodology for scanning electron microscopy (SEM) imaging for RBC morphology investigations. (PDF)

S2 Appendix Methodology for 3D confocal microscopy imaging for RBC morphology investigations. (PDF)
