# Supplementary material for: A coarse-grained red blood cell membrane model to study stomatocyte-discocyte-echinocyte morphologies
Source: PLoS One. 2019 Apr 19;14(4):e0215447. doi: 10.1371/journal.pone.0215447 (PMC6474605; doi:10.1371/journal.pone.0215447)
Supplement: S1 Appendix — (PDF) [file pone.0215447.s001.pdf]

S1 Appendix

Methodology for scanning electron microscopy (SEM) imaging for RBC morphology investigations

Ethics Approval

Ethics approval was obtained from the Blood Service Human Research Ethics Committee (Balanant270515, 27th May 2015 and Geekiyanage23062017, 23rd June 2017) and from QUT University Human Research Ethics Committee (1500000511, 9th July 2015).

RBC Samples

Fresh whole blood samples were collected in ethylenediaminetetraacetic acid (EDTA) spray-coated blood collection tubes (BD Biosciences, Franklin Lakes, New Jersey, USA) from consenting healthy volunteers. RBCs were separated from plasma by centrifugation, and used within one hour of collection. Leukodepleted packed RBC (pRBC) units were obtained from the processing department of the Australian Red Cross Blood Service (ARCBS, Kelvin Grove, Brisbane, Australia). The pRBC units were obtained from whole blood units collected into top-and-bottom bags containing citrate phosphate dextrose (Macopharma, Mouvaux, Nord, France), and processed within 24 h of collection according to standard ARCBS protocols. After centrifugation and leukoreduction, RBC were resuspended in SAGM (Macopharma).

Sample Preparation and Imaging

RBCs were sampled from 2 units of pRBCs. They were resuspended into PBS to make up 500 µL of solution at 5% haematocrit. RBCs were fixed by progressively adding 500 µL of a concentrated 2% glutaraldehyde solution into the cell suspension to reach a final concentration of glutaraldehyde of 1%. RBCs were incubated for 30 min at RT and in the dark, before being

centrifuged and washed in PBS. After fixation, 300 µL of RBC suspension at 2.5% haematocrit was adhered to coverslips coated with poly-D-lysine (Sigma-Aldrich). A second fixation step was realised on the adhered RBCs by incubating the coverslips in osmium tetroxide in cacodylate buffer (1%) for one hour (Proscitech, Kirwan, Australia). The coverslips were then dried by incubating them in an ascending series of ethanol (40-50-60-70-80-90-100%) then by incubating them with hexamethyldisilizane (HMDS, Proscitech) for 30 min. The HMDS incubation step was repeated twice before the coverslips were dried in open air for over an hour. Finally, the coverslips were gold coated and imaged with a Zeiss Sigma FESEM (Zeiss, Oberkochen, Germany).
