# Supplementary material for: A coarse-grained red blood cell membrane model to study stomatocyte-discocyte-echinocyte morphologies
Source: PLoS One. 2019 Apr 19;14(4):e0215447. doi: 10.1371/journal.pone.0215447 (PMC6474605; doi:10.1371/journal.pone.0215447)
Supplement: S2 Appendix — (PDF) [file pone.0215447.s002.pdf]

S2 Appendix

Methodology for 3D confocal microscopy imaging for RBC morphology investigations

Sample Preparation and Imaging

RBCs were washed and stained with 1,1'-dioctadecyl-3,3,3'3'-tetramethylindocarbocyanine perchlorate (DiI, 1 µL/1x10^6 cell) for 2 min at 37 °C (Thermo Fisher Scientific, Scoresby, Australia) in 200 µL 2X phosphate buffered saline (PBS, Lonza, Basel, Switzerland). After staining, the cells were fixed by progressively adding a 2% glutaraldehyde (Sigma-Aldrich, St Louis, USA) in 2X PBS until a final volume of 400 µL was reached. RBCs were incubated with glutaraldehyde for 30 min, in the dark. Concentrated PBS was used to produce echinocytes in the RBC suspension. After fixation, the cells were resuspended in 2X PBS containing 50% glycerol (Merck Millipore, Frenchs Forest, Australia) for imaging. Imaging chambers were constructed using two coverslips spaced by double-sided tape. Confocal fluorescence microscopy was performed using a Leica TCS SP5 microscope (63x 1.4NA oil) and image acquisition was realised using Leica Application Suite software (Leica, Wetzlar, Germany). Size calibration was realised using TetraSpeck fluorescent beads (Thermo Fisher Scientific, lot 1884299). Image analysis was realised using Matlab (Mathworks, Natick, USA) and a mesh of the cell surface was realised in Meshlab (Visual Computing Lab, Pisa, Italy) following the process described below.

Voxel calibration

Calibration beads were used to obtain accurate voxel size. Voxel size was calculated, using a circle and an ellipsoid fitting function [1, 2]. Calibration beads have a certified diameter of 4 µm ± 0.14 µm, it was then possible to calculate voxel dimensions and calibrate the three axes.

Image processing

After calibrating voxel size, the data obtained from RBCs were analysed and measurements extracted. The edges of the cell, corresponding to the RBC membrane, were isolated and smoothed. A number of point coordinates were selected at equally spaced intervals around the edges, forming a point cloud. All dimensions were then converted from voxel to micrometres using voxel size values obtained from the calibration beads. A homogeneous mesh was reconstituted over the point cloud to create the cell surface using Meshlab [3, 4]. The surface reconstructed using this function created a pure triangulated mesh, where each face was composed of three vertices linked together.

References

1. Bucher, I. *Circle fit (Version 1.0)*. 2005 2nd January, 2018; 29th July, 2004:[Available from: https://au.mathworks.com/matlabcentral/fileexchange/5557-circle-fit?s_tid=gn_loc_drop.
2. Petrov, Y. *Ellipsoid fit (version 1.3)*. 2015 2nd January, 2018; 04 Dec 2015:[Available from: https://au.mathworks.com/matlabcentral/fileexchange/24693-ellipsoid-fit.
3. Corsini, M., P. Cignoni, and R. Scopigno, *Efficient and Flexible Sampling with Blue Noise Properties of Triangular Meshes.* IEEE Transactions on Visualization and Computer Graphics, 2012. **18**(6): p. 914-924.
4. Kazhdan, M. and H. Hoppe, *Screened poisson surface reconstruction.* Association for Computing Machinery Transactions on Graphics, 2013. **32**(3): p. 1-13.
